# Two-Stage Gastrectomy Improves Outcomes in Perforated Gastric Cancer: A Single-Institution Retrospective Study

**DOI:** 10.7759/cureus.85002

**Published:** 2025-05-28

**Authors:** Hideo Kidogawa, Nobutaka Matayoshi, Takeshi Konno, Takashi Okimoto, Toshihito Uehara, Junya Noguchi, Takatomo Yamayoshi, Shin Shinyama, Satoshi Kimura, Kohji Okamoto

**Affiliations:** 1 Department of Surgery, Kitakyushu City Yahata Hospital, Kitakyushu, JPN; 2 Department of Clinical Pathology, Kitakyushu City Yahata Hospital, Kitakyushu, JPN

**Keywords:** gastrectomy, laparoscopic surgery, omental patch repair, perforated gastric cancer, two-stage surgery

## Abstract

Background: Perforated gastric cancer (PGC) is a rare but life-threatening surgical emergency. Due to its low reported incidence and significant challenges in the preoperative diagnosis of malignancy, there is no established consensus on optimal management. Recent evidence suggests that a two-stage surgical strategy, involving initial damage control followed by delayed curative resection, may reduce morbidity and mortality while improving oncological outcomes. Preoperative diagnosis of malignancy is uncommon, and the utility of routine intraoperative biopsy has been questioned, highlighting the importance of postoperative endoscopic biopsy for definitive diagnosis. This study aims to evaluate the safety and effectiveness of a two-stage surgical strategy for PGC, emphasizing its adaptability and outcomes based on a 27-year retrospective analysis at a single institution.

Methods: We retrospectively reviewed 62 cases of gastric perforation treated between 1998 and October 2023. Among them, nine cases were pathologically confirmed as PGC. Initial management involved conservative therapy or omental patch repair (via laparotomy or laparoscopy) aimed at stabilizing the patient's condition and managing peritonitis. Following malignancy confirmation (often by postoperative biopsy) and clinical stabilization, elective curative gastrectomy with appropriate lymphadenectomy was planned for eligible patients. Postoperative outcomes, including complications (graded by Clavien-Dindo), curative resection rates, and survival, were analyzed.

Results: Of the nine PGC patients (median age 74 years), none were diagnosed preoperatively. Initial management included conservative therapy (n=3), open omental patch repair (n=3), and laparoscopic omental patch repair (n=3). No Clavien-Dindo grade III or higher complications were observed following initial management. Eight patients subsequently underwent gastrectomy, with seven achieving R0 resection. One patient succumbed to cancer progression during hospitalization prior to the planned gastrectomy. For the seven patients who underwent R0 resection, the median follow-up was 24 months (range: 12-56 months). At the final data cut-off (April 2025), three of these seven R0 patients were alive and disease-free; one had died from cancer recurrence, and three were lost to follow-up while alive. Notably, one complex case with initial peritoneal dissemination (Case 6) successfully underwent delayed radical resection after chemotherapy, illustrating the strategy's adaptability. Conversely, Case 7 exemplified a straightforward and successful two-stage laparoscopic approach, resulting in long-term disease-free survival. This patient is one of the three currently alive.

Conclusion: This retrospective study suggests that a two-stage surgical strategy, involving initial stabilization followed by elective curative gastrectomy, is a safe and effective approach for managing PGC. It achieved high rates of R0 resection with low morbidity and demonstrated promising survival outcomes in this high-risk cohort. Further multicenter prospective studies are needed to establish standardized protocols and validate patient selection criteria.

## Introduction

Gastric perforation is most commonly caused by benign ulcers, but malignant etiologies, including gastric cancer, are occasionally encountered. The reported incidence of gastric cancer among perforated gastric cases varies widely in the literature, with reported rates ranging from 4.2% to 40%, based on retrospective clinical studies [1-8]. Preoperative differentiation between benign and malignant perforation remains challenging, as clinical presentations are often similar [6,9-11]. Traditionally, emergency gastrectomy has been the standard approach; however, recent studies suggest that a two-stage surgical strategy, initial stabilization followed by delayed curative resection, may reduce morbidity and mortality while improving oncological outcomes [7,12-15]. Despite emerging evidence, there is no standardized treatment protocol for perforated gastric cancer (PGC). At our institution, we have adopted a strategy of conservative or minimally invasive management in the acute phase, followed by curative gastrectomy if malignancy is confirmed and the patient's condition permits. Herein, we retrospectively analyze our institutional experience with this approach.

## Materials and methods

This retrospective study was conducted at Kitakyushu City Yahata Hospital in Kitakyushu, Japan. We reviewed the medical records of patients who presented with gastric perforation and were treated at our institution between January 1998 and October 2023.

Patient selection

A total of 62 consecutive cases of gastric perforation were identified during the study period. Among these, the inclusion criterion for this study was a pathological diagnosis of PGC. Based on this criterion, nine patients were included in the analysis. Patients diagnosed with gastric perforation due to benign causes were excluded, as the focus of this study was solely on PGC. Data from patients with incomplete medical records or insufficient follow-up information were also excluded from the analysis.

Data collection

Clinical data were retrospectively collected from electronic and paper medical records. The variables collected included patient demographics (age, sex), initial diagnostic method (e.g., imaging, endoscopy), initial treatment approach (conservative therapy, laparoscopic omental patch repair, open omental patch repair), details of surgical procedures performed (type of gastrectomy, lymphadenectomy), pathological findings (TNM staging according to the Union for International Cancer Control (UICC) staging system, tumor differentiation), postoperative complications, and clinical outcomes (hospital stay, survival status, follow-up duration).

Treatment protocol

Initial management strategies for gastric perforation varied depending on the patient's clinical condition and the suspected etiology. Initial management involved either conservative therapy (including nasogastric tube drainage, antibiotics, and fluid resuscitation) or surgical closure of the perforation using either laparoscopic or open omental patch repair. The primary goal of initial treatment was patient stabilization and management of peritonitis. Following the acute phase and confirmation of malignancy through biopsy (either intraoperative or postoperative endoscopy), a staged approach was considered. Elective curative gastrectomy with appropriate lymphadenectomy was planned for patients who were clinically stable and deemed to have resectable disease. The decision to proceed with staged gastrectomy was made on a case-by-case basis, considering the patient's overall condition and the extent of the cancer.

Outcome evaluation

Postoperative complications within 30 days after each surgical procedure were graded according to the Clavien-Dindo classification system. Long-term survival outcomes were assessed based on follow-up data from medical records and communication with patients or their families.

Statistical analysis

This study is primarily descriptive due to the small sample size. Clinical characteristics and outcomes are presented using descriptive statistics, including median values and ranges where appropriate. Survival duration was calculated from the date of the initial treatment. Follow-up was censored as of April 2025.

## Results

Among the nine patients with PGC, the median age was 74 years. Notably, none were diagnosed with gastric cancer prior to surgery. Initial management included conservative therapy in three patients, open omental patch closure in three, and laparoscopic omental patch closure in the remaining three. No major postoperative complications (Clavien-Dindo grade III or higher) were observed in any group.

**Figure 1:**
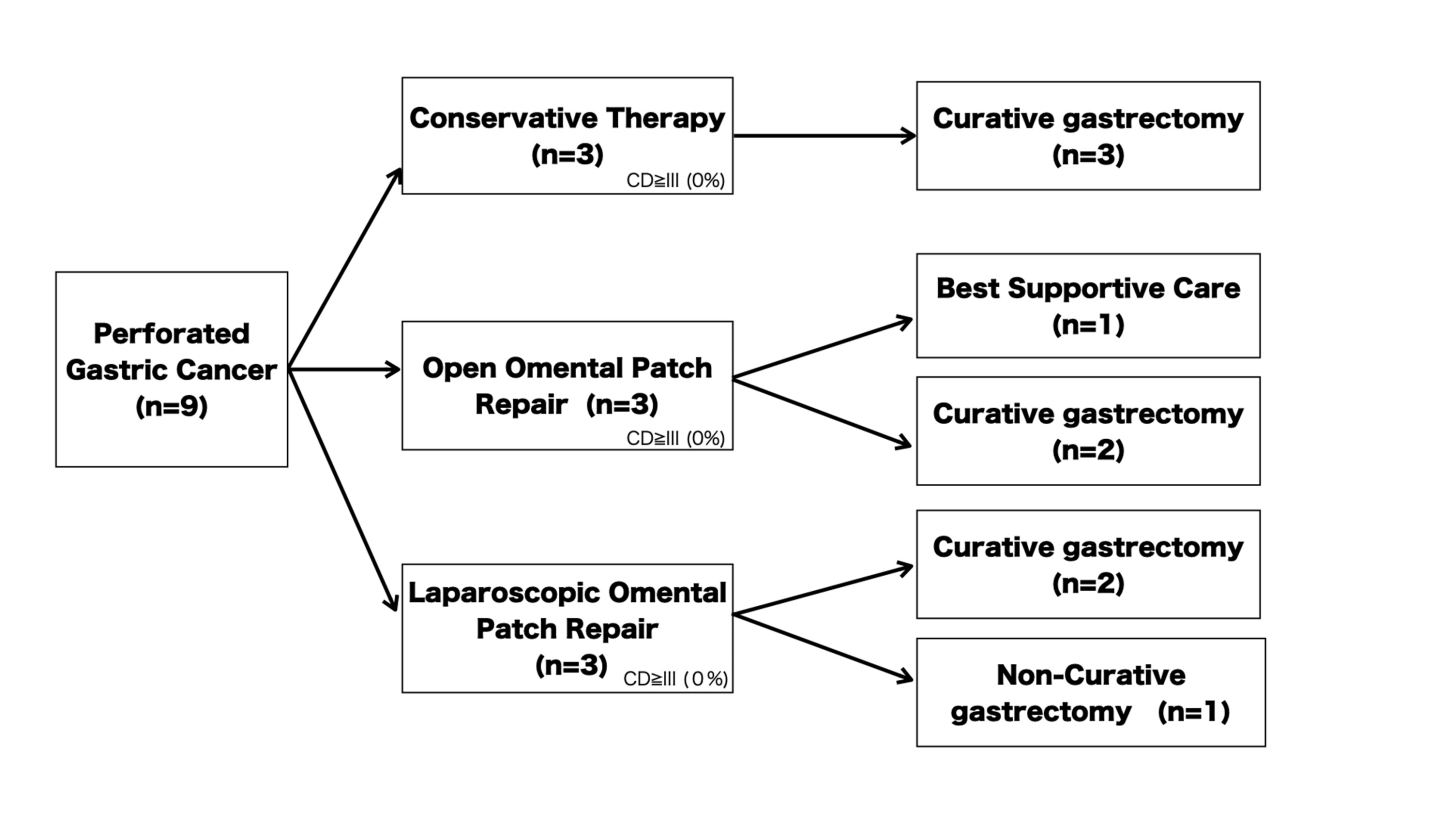
Clinical course and surgical management of nine patients with perforated gastric cancer. Initial treatment included conservative management or omental patch repair (laparoscopic or open). Seven patients subsequently underwent gastrectomy with curative R0 resection. Of these seven R0 resected patients, three remain alive and disease-free, one died of cancer, and three were lost to follow-up while alive (see text and Table 1 for details on follow-up duration and specific outcomes). The figure illustrates treatment pathways and outcomes.

Eight patients subsequently underwent gastrectomy. Of these, seven underwent curative R0 resection, while one patient underwent a non-curative resection due to advanced disease. One patient received best supportive care following initial omental patch closure and did not proceed to definitive surgery. The overall clinical course and surgical management of the nine patients are summarized in Figure 1.

No major postoperative complications (Clavien-Dindo classification grade III or higher) were observed in any patient. One patient died during the hospital stay due to the rapid progression of the underlying malignancy. For the five patients who underwent an initial surgical repair for perforation followed by a subsequent gastrectomy, the median time interval between these two operations was 32 days (mean: 67 days; range: 20-214 days). Seven patients underwent curative R0 resection. For these seven patients, the overall follow-up period ranged from 12 to 56 months, with a median follow-up of 24 months (data censored as of April 2025). As detailed in Table 1, three of these patients were alive and disease-free at their last follow-up (with follow-up durations of 19, 24, and 54 months, respectively). One patient died from cancer recurrence 56 months postoperatively. The remaining three patients were lost to follow-up while alive at 12, 23, and 42 months, respectively. Notably, Case 6 underwent three separate surgeries. Initially, laparoscopic omental patch repair was performed to manage the perforation. During the second surgery, an attempted laparoscopic gastrectomy revealed peritoneal dissemination, leading to palliative bypass surgery instead. Following chemotherapy, complete resolution of peritoneal metastasis was confirmed, allowing successful laparoscopic gastrectomy during the third operation. The patient remains disease-free with no recurrence, highlighting the potential of staged surgical management combined with systemic therapy in advanced cases.

**Table 1:** Summary of clinical characteristics, surgical management, and outcomes of nine patients with perforated gastric cancer (PGC). Among the nine PGC patients, seven underwent R0 resection. For these seven R0 resected patients, the median follow-up was 24 months (range 12-56 months). Three patients remain alive and disease-free, one died of cancer at 56 months, and three were lost to follow-up while alive (at 12, 23, and 42 months, respectively).

Case	Age	Sex	Initial Diagnosis	Definitive Diagnosis	Initial Treatment	2nd Treatment	Tumor Location	Stage	Prognosis
1	74	M	Perforated gastric ulcer	Postoperative endoscopic biopsy	Open omental patch closure	Open distal gastrectomy	Gastric angle, lesser curvature	pT3N0M0 Stage IIA	Alive at last follow-up (12 months)
2	88	F	Gastrointestinal perforation	Intraoperative biopsy (postop diagnosis)	Open omental patch closure	Best supportive care	Antrum, anterior wall	cT3N1M0 Stage IIB	88 days, Death due to cancer progression
3	60	M	Perforated gastric ulcer	Endoscopic biopsy	Conservative therapy	Open distal gastrectomy	Antrum, lesser curvature	cT3N2M0 Stage IIIA	Alive at last follow-up (23 months)
4	71	M	Perforated gastric cancer	Intraoperative biopsy (postop diagnosis)	Open omental patch closure	Open distal gastrectomy	Antrum, anterior wall	pT3N2M0 Stage IIIA	56 months, Death due to cancer progression
5	79	M	Perforated gastric ulcer	Endoscopic biopsy	Conservative therapy	Open total gastrectomy	Mid-body, lesser curvature	pT3N2M0 Stage IIIA	Alive at last follow-up (42 months)
6	70	M	Perforated gastric ulcer	Postoperative endoscopic biopsy	Laparoscopic omental patch closure	1) Laparoscopic gastrojejunal bypass; 2) Laparoscopic distal gastrectomy	Gastric angle, lesser curvature	pT3N2M0 Stage IIIA	Alive at last follow-up (54 months)
7	62	M	Perforated gastric ulcer	Postoperative endoscopic biopsy	Laparoscopic omental patch closure	Laparoscopic distal gastrectomy	Gastric angle, lesser curvature	pT4N2M0 Stage IIIB	Alive at last follow-up (24 months)
8	75	F	Perforated gastric ulcer	Endoscopic biopsy	Conservative therapy	Open total gastrectomy	Upper body, lesser curvature	pT4bN2M0 Stage IIIB	Alive at last follow-up (19 months)
9	96	F	Perforated gastric ulcer	Postoperative endoscopic biopsy	Laparoscopic omental patch closure	Open total gastrectomy	Mid-body, lesser curvature	pT4aN1M0 Stage IIIA	Alive at last follow-up (16 months)

Representative case

**Figure 2:**
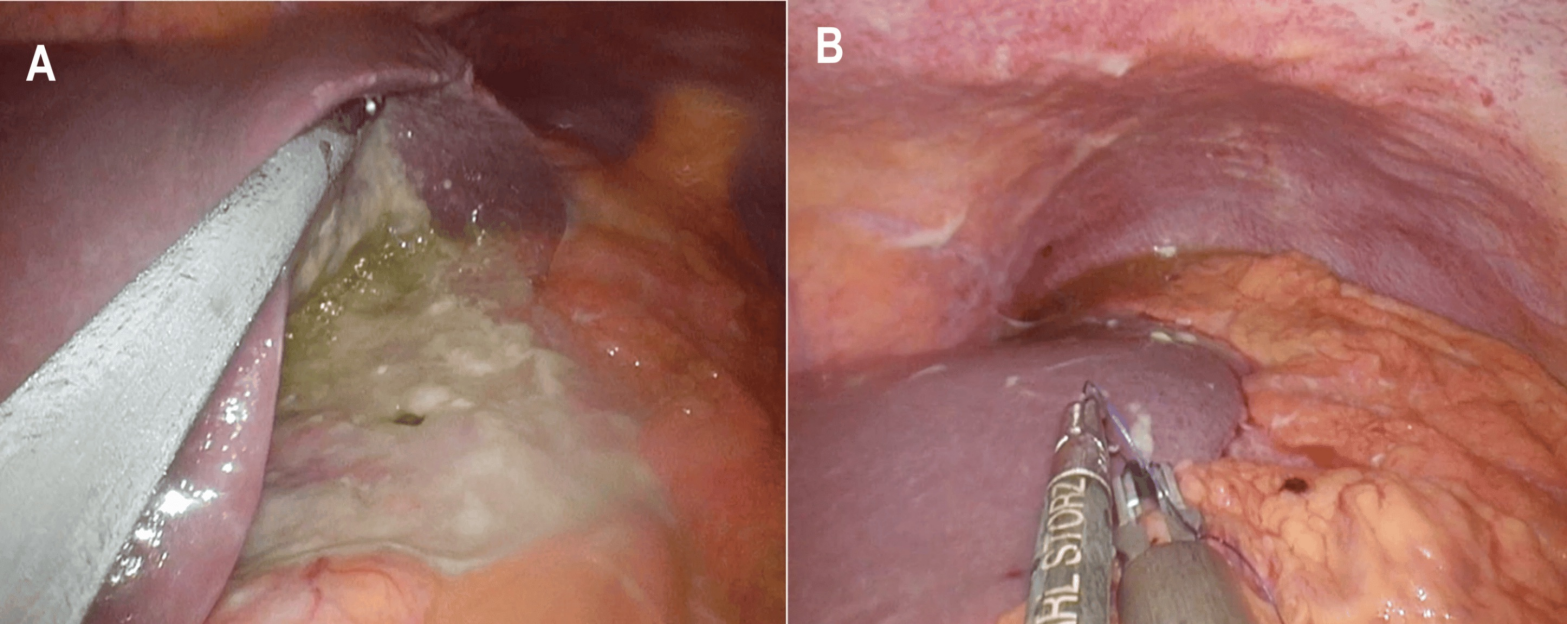
Laparoscopic omentoplasty performed for gastric perforation (Case 7). (A) Intraoperative view showing purulent contamination around the perforation site during laparoscopic exploration. (B) Omental patch being sutured over the perforation site to complete closure.

**Figure 3:**
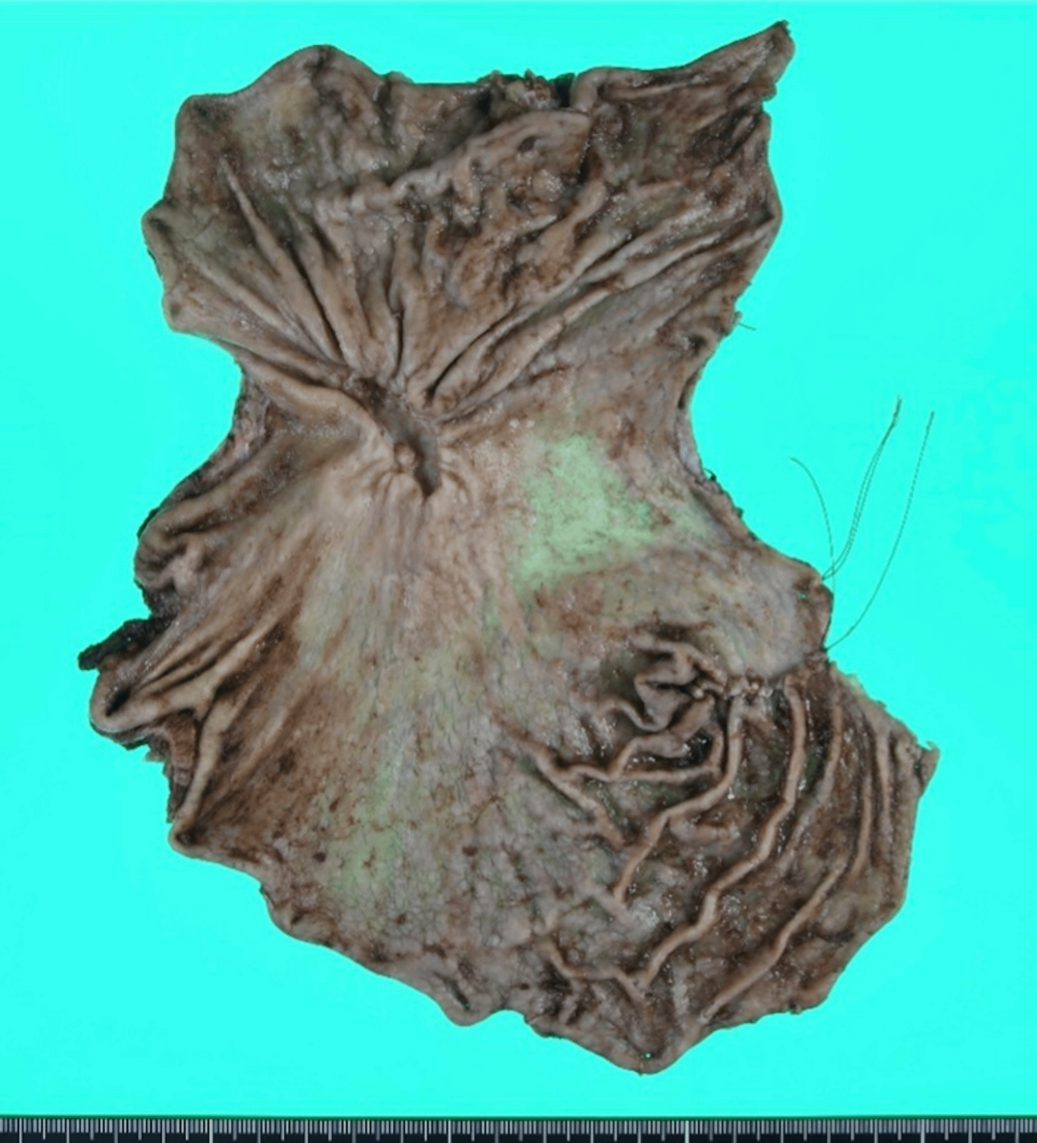
Resected stomach specimen showing a type 3 tumor located at the gastric angle (Case 7). The lesion measures 90×50 mm and exhibits macroscopic features such as ulceration and apparent infiltration.

**Figure 4:**
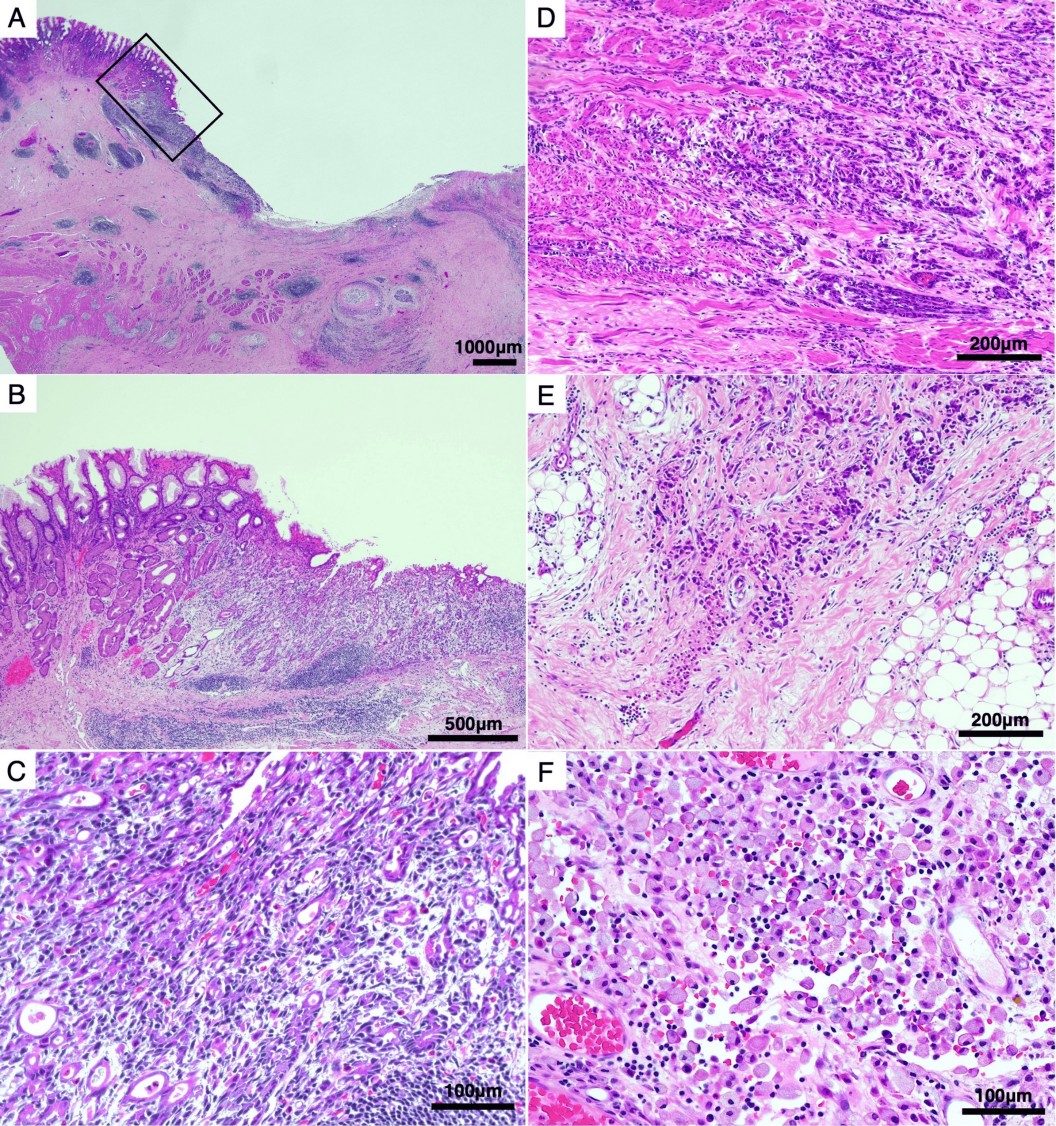
Histological progression from mucosal adenocarcinoma to poorly differentiated and signet ring cell components (Case 7). (A) Low-power view of the gastric tumor showing an ulcerative lesion. (B) Intermediate magnification of the ulcer ridge (corresponding to the black box in A). (C) High-power view of moderately differentiated adenocarcinoma confined to the mucosa. (D, E) Invasive tumor components extending into the muscularis propria (D) and serosa (E), composed predominantly of poorly differentiated adenocarcinoma. (F) High-power view revealing scattered signet ring cell carcinoma components in the invasive area.

This case, which corresponds to Case 7 in Table 1, describes a 62-year-old man who presented with sudden-onset upper abdominal pain. His medical history included hypertension and type 2 diabetes mellitus. Abdominal computed tomography (CT) revealed the presence of free air in the peritoneal cavity, indicating gastrointestinal perforation. Emergency upper gastrointestinal endoscopy identified a large ulcer at the gastric angle. Although malignancy could not be definitively excluded, initial findings suggested the need for surgical intervention. The patient underwent laparoscopic single-port omental patch repair with extensive peritoneal lavage (Figure 2). Postoperative recovery was uneventful, and endoscopic biopsy performed during the stabilization phase confirmed moderately to poorly differentiated adenocarcinoma. Fifty days after the initial surgery, the patient underwent a laparoscopic distal gastrectomy with D2 lymphadenectomy, achieving R0 resection. The resected stomach specimen revealed a type 3 tumor located at the gastric angle, measuring 90 x 50 mm (Figure 3). Histopathological analysis confirmed moderately to poorly differentiated adenocarcinoma, classified as pT4aN2M0 (Stage IIIB), with invasive components extending into the muscularis propria and serosa, and scattered signet ring cells (Figure 4). At 24 months postoperatively, the patient remains alive and disease-free, highlighting the potential effectiveness of the staged surgical strategy for advanced gastric cancer with perforation.

## Discussion

PGC is an uncommon but critical emergency, occurring in fewer than 1% of all gastric malignancies [7]. The incidence of gastric cancer among perforated gastric cases varies widely in the literature, reflecting differences in study populations and methodologies, with reported rates ranging from 4.2% to 40%, based on retrospective clinical studies [1-8]. Preoperative differentiation between benign and malignant perforation remains challenging, as clinical presentations are often similar [6,9-11]. Traditionally, emergency gastrectomy has been the standard approach; however, recent studies suggest that a two-stage surgical strategy, initial stabilization followed by delayed curative resection, may reduce morbidity and mortality while improving oncological outcomes [7,12-15]. Despite emerging evidence, there is no standardized treatment protocol for perforated gastric cancer. At our institution, we have adopted a strategy of conservative or minimally invasive management in the acute phase, followed by curative gastrectomy if malignancy is confirmed and the patient's condition permits. Herein, we retrospectively analyze our institutional experience with this approach.

Our series also illustrates the adaptability of staged management. While most patients followed a straightforward two-stage protocol exemplified by Case 7, one patient (Case 6) presented a complex scenario requiring multiple interventions and systemic chemotherapy before achieving radical resection. This highlights the flexibility required in managing PGC, particularly in advanced stages, and demonstrates that temporary non-curative management can be safely converted to radical resection following effective systemic therapy. This flexible approach, mirroring findings by Terayama et al. [16] who demonstrated the feasibility of "stomach-preserving strategies" in patients with distant metastasis, aligns with strategies aiming for tumor downstaging before definitive surgery in select cases. Conversely, Case 7 typifies straightforward two-stage care: omentoplasty (Figure 2) was followed 50 days later by laparoscopic D2 distal gastrectomy for pT4aN2M0 disease (Figures 3, 4), and the patient has remained recurrence-free for 22 months. The successful long-term survival observed in this advanced-stage patient is noteworthy, as long-term survival after perforation of advanced gastric cancer is generally considered rare [17].

This structured, staged approach is consistent with the work of Kim et al. [13], who reported a five-year disease-free survival of 80% after laparoscopic two-stage management, emphasizing the benefit of delayed resection in stabilizing oncological control and improving surgical precision. Similar staged approaches and analyses of treatment outcomes in cohorts of PGC patients have been reported by other centers [5,15], contributing valuable insights into the management of this challenging condition. The flexibility to adapt surgical strategy based on intraoperative findings and patient response to chemotherapy reflects the growing trend towards personalized surgical planning in gastric cancer management, balancing emergency needs with oncological principles [8]. Furthermore, the role of minimally invasive approaches in managing perforated gastric cancer has been emphasized in recent literature. Melloni et al. [18] conducted a systematic review demonstrating the safety and effectiveness of laparoscopic interventions in the emergency setting, highlighting their potential to reduce perioperative complications and improve recovery times. Di Carlo et al.'s [8] recent critical appraisal also supports the potential utility of laparoscopic simple closure as a first step in the staged management of PGC. These findings suggest that a laparoscopic two-stage strategy could further enhance outcomes in this high-risk patient population.

**Figure 5:**
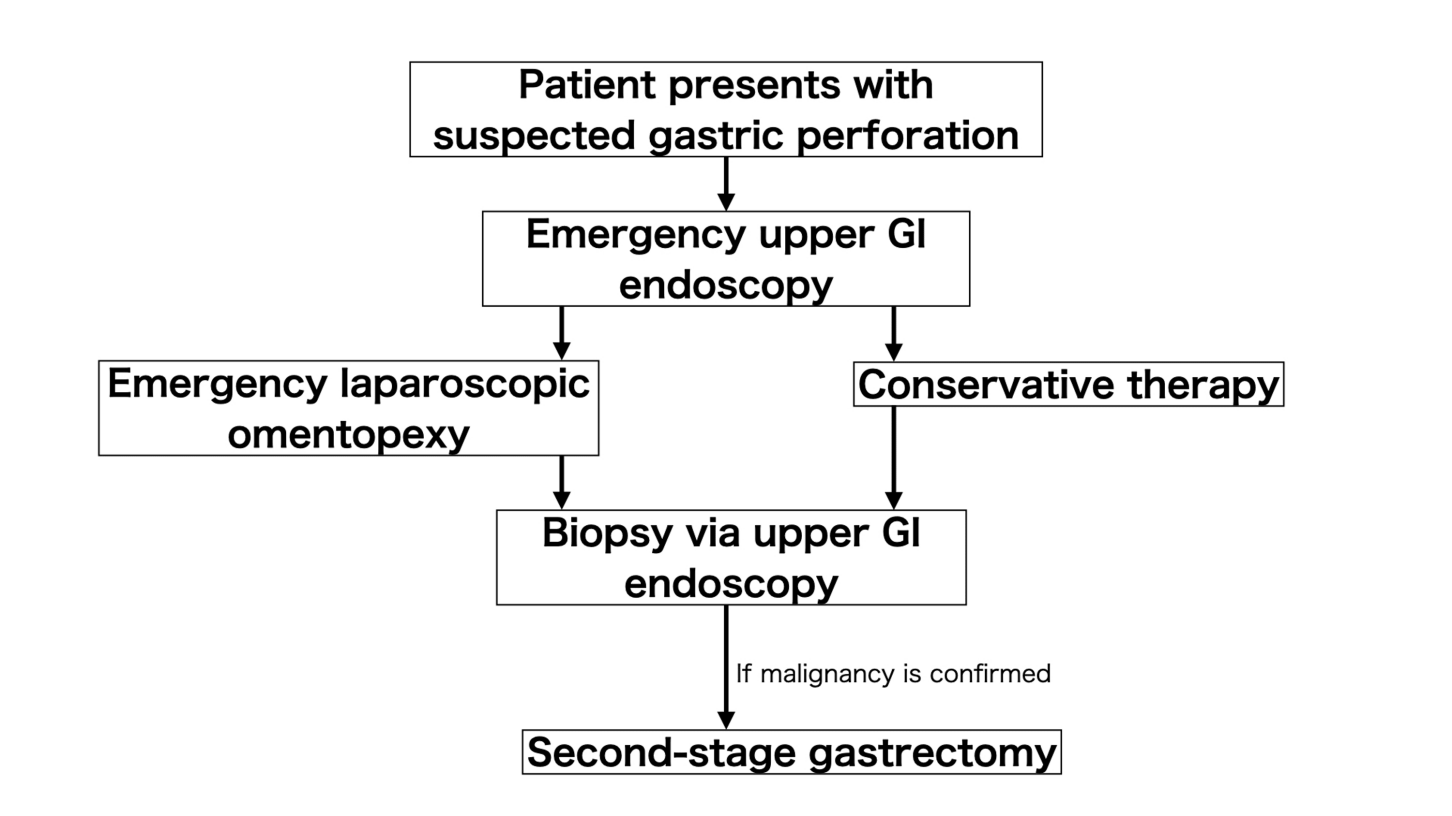
Institutional treatment algorithm for gastric perforation. This flowchart illustrates our institutional approach to the management of gastric perforation. The protocol involves early upper GI endoscopy for diagnostic clarification, emergency laparoscopic omental patch repair when indicated, and biopsy during the recovery phase. If malignancy is confirmed, second-stage gastrectomy is planned. Conservative therapy may be considered for stable patients with sealed perforation and no evidence of malignancy.

Although preoperative diagnosis of malignancy remains challenging (none of our patients were diagnosed before the first surgery), this difficulty is well recognized, with reported detection rates below 40% [4,9]. Leeman et al. [6] reported similar diagnostic challenges in their series, emphasizing the importance of follow-up endoscopy for definitive diagnosis, as malignancy was sometimes only identified during the recovery phase. Furthermore, the utility of routine intraoperative biopsy has been questioned in recent literature; a prospective study by Steyn et al. [11] suggested that delayed endoscopic biopsy is a more reliable approach to confirm or exclude malignancy in gastric perforations. The staged pathway, as implemented in our practice, addresses this diagnostic dilemma by permitting biopsy confirmation during the recovery interval, accurate staging, and subsequent incorporation of perioperative chemotherapy when indicated. This stepwise approach ensures that malignancy is not overlooked while stabilizing the patient's condition before definitive resection. Our institutional algorithm (Figure 5) formalizes this approach, recommending early endoscopy for initial assessment, laparoscopic closure in unstable patients, biopsy during convalescence, and elective D2 gastrectomy if malignancy is confirmed. Conservative therapy is reserved for stable patients with sealed perforation and no radiological or clinical evidence of cancer, reflecting a selective strategy aimed at optimizing patient outcomes while minimizing unnecessary surgical risks.

This study is limited by its retrospective design, small sample size, and single-center setting. These limitations are commonly encountered in studies of PGC due to its rarity, highlighting the challenges in accumulating large cohorts [8]. Despite these limitations, the consistency of our outcomes and the absence of major complications observed in our series support the real-world feasibility and safety of the two-stage strategy. Further multicenter prospective studies are required to validate selection criteria, determine optimal timing for second-stage gastrectomy, and clarify the role of perioperative therapy, including chemotherapy and immunotherapy. As highlighted in recent comprehensive reviews [8], future research should also focus on improving preoperative diagnosis, clarifying the optimal surgical approach (including the role of minimally invasive techniques), and investigating strategies for preventing peritoneal dissemination. The difficulty in preoperatively diagnosing PGC was underscored in our series, with no definitive preoperative malignant diagnoses. Our institutional priority in these emergencies is immediate patient stabilization and peritonitis control, rather than definitive etiological diagnosis. Thus, initial non-contrast CT primarily assessed perforation severity, not detailed oncological features. Even when one radiology report (Case 9) post-operatively noted CT findings suspicious for malignancy (e.g., irregular wall thickening, lymphadenopathy), the emergent surgical intervention had already proceeded based on a presumptive benign diagnosis focused on life-saving measures. The remaining eight cases lacked specific CT signs of malignancy. This approach, prioritizing stabilization over immediate definitive diagnosis, is central to our two-stage strategy, acknowledging emergency diagnostic limitations and aiming to optimize patients for potential oncological surgery post-confirmation. Nonetheless, the present data, together with contemporary literature [7,10,12-15], indicate that a two-stage surgery should generally be considered the default strategy for most patients with suspected PGC, particularly the elderly or those presenting with sepsis, where initial stabilization is paramount [14,15]. One-stage gastrectomy may still be appropriate for highly selected patients, such as young, hemodynamically stable individuals with a definitive intra-operative diagnosis and minimal peritoneal contamination [19]. However, for the majority, staged management safely bridges emergency stabilization and oncologically sound surgery, providing a pathway toward achieving adequate lymph-node harvest, lower perioperative morbidity, and improved disease-free survival, as supported by our series and published data [7,12-15,17,20].

## Conclusions

Perforated gastric cancer is a rare and high-risk surgical emergency. Our experience suggests that initial minimally invasive control of perforation, followed by elective gastrectomy after stabilization, is feasible and effective. This two-stage strategy has achieved high rates of R0 resection with low morbidity, demonstrating favorable long-term survival. Multicenter prospective studies are needed to establish standardized treatment protocols and validate patient selection criteria.
